# Global phylogenetic analysis of contemporary aleutian mink disease viruses (AMDVs)

**DOI:** 10.1186/s12985-017-0898-y

**Published:** 2017-11-22

**Authors:** P. Ryt-Hansen, E. E. Hagberg, M. Chriél, T. Struve, A. G. Pedersen, L. E. Larsen, C. K. Hjulsager

**Affiliations:** 1 National Veterinary Institute, Technical University of Denmark, Bülowsvej 27, DK-1870 Frederiksberg C, Denmark; 2 Kopenhagen Fur, Langagervej 60, DK-2600 Glostrup, Denmark; 3 Center for Biological Sequence Analysis, Technical University of Denmark, Kemitorvet Building 208, DK-2800 Lyngby, Denmark

**Keywords:** Aleutian mink disease virus, Parvovirus, NS1, Molecular characterization, Phylogenetics, Bayesian tree

## Abstract

**Background:**

Aleutian mink disease has major economic consequences on the mink farming industry worldwide, as it causes a disease that affects both the fur quality and the health and welfare of the mink. The virus causing this disease is a single-stranded DNA virus of the genus *Amdoparvovirus* belonging to the family of *Parvoviridae*. In Denmark, infection with AMDV has largely been restricted to a region in the northern part of the country since 2001, affecting only 5% of the total Danish mink farms. However, in 2015 outbreaks of AMDV were diagnosed in all parts of the country. Initial analyses revealed that the out breaks were caused by two different strains of AMDV that were significant different from the circulating Danish strains. To track the source of these outbreaks, a major investigation of global AMDV strains was initiated.

**Methods:**

Samples from 13 different countries were collected and partial NS1 gene was sequenced and subjected to phylogenetic analyses.

**Results:**

The analyses revealed that AMDV exhibited substantial genetic diversity. No clear country wise clustering was evident, but exchange of viruses between countries was revealed. One of the Danish outbreaks was caused by a strain of AMDV that closely resembled a strain originating from Sweden. In contrast, we did not identify any potential source for the other and more widespread outbreak strain.

**Conclusion:**

To the authors knowledge this is the first major global phylogenetic study of contemporary AMDV partial NS1 sequences. The study proved that partial NS1 sequencing can be used to distinguish virus strains belonging to major clusters. The partial NS1 sequencing can therefore be a helpful tool in combination with epidemiological data, in relation to outbreak tracking. However detailed information on farm to farm transmission requires full genome sequencing.

**Electronic supplementary material:**

The online version of this article (10.1186/s12985-017-0898-y) contains supplementary material, which is available to authorized users.

## Background

Aleutian mink disease virus (AMDV) is a single stranded DNA virus with a genome length of 4.8 kb. AMDV belongs to the family *Parvoviridae* [1]*,* genus *Amdoparvovirus*. Compared to other DNA viruses, parvoviruses have relatively high mutation rates similar to that of RNA viruses [2, 3].

AMDV is one of the most important pathogens in mink farming worldwide. In adults, the virus causes plasmacytosis, an immune-mediated disease where an excessive expantion of plasma cells and subsequent production of antibodies leads to systemic accumulation of immune complexes. The infection often becomes chronic in adults leading to impaired animal welfare, reproduction and pelt quality. The kits on the other hand display an acute form of the disease, as they can develop interstitial pneumonia leading to respiratory distress with high mortality [1, 4, 5]. Aleutian mink disease was initially thought to be a hereditary disease limited to the specific genotype of Aleutian mink. Later it was revealed that the conditions were caused by a virus and the disease is not limited to the Aleutian genotype [1]. It has been hypothesized that the disease originated in North America, and spread to Europe when American mink were imported in the1920–1930s [6, 7].

Aleutian mink disease is enzootic in most countries, and only Iceland has been successful in eradicating the virus from farmed mink [8]. In 1976, Denmark initiated a voluntary control program and in 1999 legislation was implemented to make all farms test their breeders for the purpose of establishing the disease status of the farm. This eradication program resulted in only 5% of Danish farms being positive for AMDV in 2001, with all positive farms being restricted to one specific region in the northern part of the Jutland peninsula. The circulating viruses in this part of the country all show a very high degree of sequence homology and are referred to as the Saeby strain [9]. Another distinct strain of AMDV was discovered to be circulating in the wild minks of Bornholm, but it has so far stayed restricted to the island [10]. In 2015, however, outbreaks of AMDV appeared affecting all regions of Denmark with unknown source.

The partial NS1 sequencing of viruses from this outbreak revealed that the outbreaks were caused by two distinct viral strains, which clearly deviated from all viruses involved in previously known outbreaks in Denmark [11]. Furthermore, there was only limited nucleotide sequence homology to any AMDV sequences in GenBank. Specifically, the partial NS1 sequences showed the presence of two new virus clusters; one named the Zealand cluster, as the viruses were all from farms located on Zealand, and the other named the “Holstebro” cluster containing viruses isolated from farms in both Jutland and Funen and with index case from the town Holstebro [11]. In order to investigate the possible origin of these new Danish outbreaks we collected AMDV strains from other countries with commercial mink production. The partial NS1 gene was sequenced and subjected to phylogenetical analysis. The PCR targeting the NS1 gene was chosen since it was an already established method that proved robust and allowed us to obtain sequences fast, which was crucial in an outbreak situation. Furthermore the majority of previous studies within the field have used the same method which allowed for comparison with sequences generated in earlier studies [9–12].

## Methods

### Samples

Veterinarians and farmers in countries with mink production were encouraged to contribute with samples for the project. The aim was to sample 10 farms from each country. The animals were chosen by the veterinarian or the farmer based on clinical signs. Mink carcasses or blood and spleen samples were submitted to the Danish National Veterinary Institute. In total, more than 525 animals were sampled from 13 countries: Canada, Denmark, Finland, Greece, the Netherlands, Iceland, Italy, Latvia, Lithuania, Poland, Sweden, Spain and USA. All samples were obtained with the farmer’s consent, and donated to the project for research. An overview of samples received and sequenced from each country is presented in Table 1. A blood sample from the heart and spleen were collected from each carcass. In addition, blood, spleen and mesenteric lymph node samples from Danish mink farms were supplied by Kopenhagen Fur. From each farm, five animals were tested by PCR, and up to three positive samples from each farm were sequenced. All blood samples were tested for AMDV antibodies using ELISA [13] and counterimmunoelectrophoresis at Kopenhagen Diagnostics (Kopenhagen Fur, Glostrup, Denmark). Samples from 5 farmed and one wild Danish mink in addition to one Norwegian farmed mink submitted for routine diagnostic examination at the National Veterinary Institute during 2015–2016 were also included in the study.

**Table 1:** Overview of the samples included in the study

Country:	No. of sampled animals:	No. of sampled farms:	No. of obtained sequences:
Poland	42	8	23
Lithuania	12	3	8
Finland	62	13	38
Latvia	5	1	3
USA	24	6	16
Canada	50	11	29
The Netherlands	60	12	36
Italy	25	5	13
Greece	40	9	22
Iceland	16	0	4
Sweden	34	7	21
Denmark	162	73	137

### DNA extraction, PCR and sequencing

Total DNA was extracted using the QIAmp®DNA Mini Kit with the Standard version 1 protocol automated on the QIAcube (Qiagen, Copenhagen, Denmark). PCR and Sanger sequencing was carried out as previously described [11]. The sequences names were tagged “f” or “w” to indicate if the mink was farmed or wild respectively, and included the country and region of sampling, and the sampling date as follows: AMDV_mink-f/w_Country_unique identifier_sampling date. Samples from the routine diagnostics were named with prefix “Diag”.

### Sequencing and phylogenetic analyses

The nucleotide sequences were analyzed with CLC main workbench version 7.5 (https://www.clcbio.com, QIAGEN, Aarhus, Denmark). For each sample, sequence chromatogram files resulting from the forward and reverse primers were contiged and manually proof-read. The primer binding regions [14] were trimmed off to generate 328 bp consensus sequences from each sample. Consensus sequences were aligned at the nucleotide level with existing partial NS1 sequences in GenBank NCBI retrieved in June 2016, using the “MUSCLE” multiple alignment algorithm implemented in CLC. Initially an alignment of all AMDV partial NS1 gene sequences available in GenBank was created, and then representative sequences of each cluster was included in the final alignment. This reduction was done merely to reduce the number of reference sequences in the final tree to facilitate interpretation of the tree. The percentage sequence identity and number of nucleotide differences among pairs of sequences was determined using the “pairwise comparison” functionality implemented in the CLC software. The software jModelTest version 2.1.10 [15] was applied to identify the general time reversible model with gamma-distributed rates (GTR + G) as the best fitting substitution model. The phylogeny was reconstructed using Bayesian approach with Markov Chain Monte Carlo (MCMC) sampling implemented in MrBayes v.3.2.5 [16]. The analysis was run for 10,000,000 generations with samples drawn every 1000 generations, and convergence was verified by ensuring that two parallel sampling chains converged to similar posterior distributions. The final tree was visualized and manipulated in FigTree version 1.4 (http://tree.bio.ed.ac.uk/software/figtree/).

## Results

**Fig. 1:**
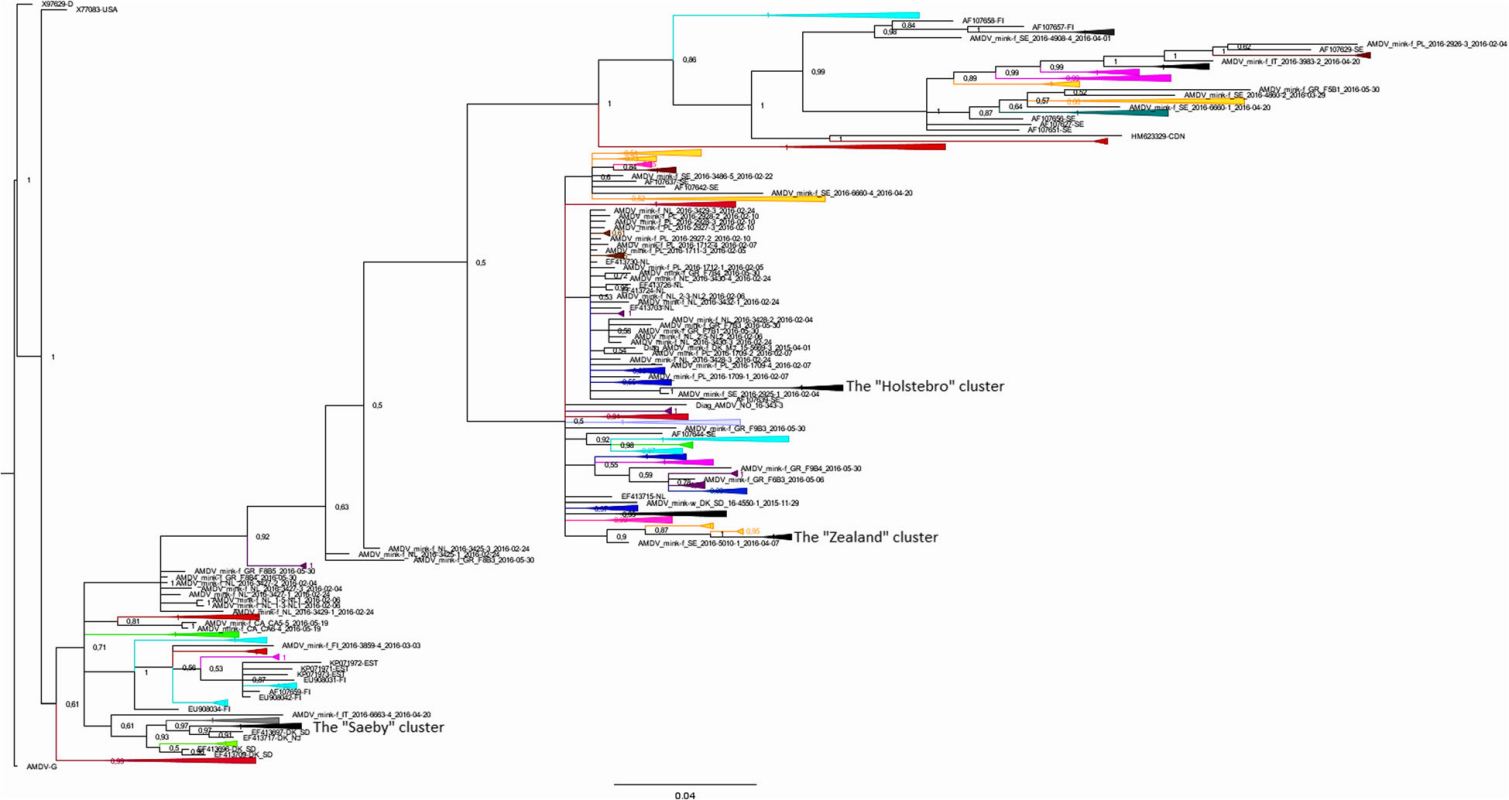
Bayesian tree of the global distribution of partial NS1 sequences. Color coding of the tree given by the clusters: brown: Poland, dark green: Iceland, green: USA, turquois: Finland, blue: The Netherlands, purple: Italy, yellow: Estonia, grey: Latvia, lavender: China, orange: Sweden, pink: Lithuania, red: Canada, dark grey: Spain, aubergine color: Greece and black: Denmark. Single sequences outside clusters are named as mentioned in the text: AMDV_mink-f/w_country code_unique identifier_sampling date. “f” or “w” indicates if the mink was farmed or wild. Samples from the routine diagnostics were named with prefix “Diag”. Sequences from GenBank are named with their accession number and country code as suffix. A detailed tree can be found in the Additional file 1

The pairwise sequence identity when comparing the aligned partial NS1 sequences ranged from 78% to 100% corresponding to between 0 and 73 differing nucleotide positions. The phylogenetic tree reconstructed from the partial NS1 alignment is shown in Fig. 1. Further details can be found in the Additional file 1.

Overall, viral sequences originating from the same country did not cluster together, but tended to be dispersed around the tree while sequences originating from the same farm most often clustered together and had pairwise sequence identities in the range of 96% to 100%. However, some farms exhibited high within farm diversity. For instance the sequences named AMDV_mink-f_IT_2016–6663-3 and AMDV_mink-f_IT_2016–6663-5 originated from different minks from the same farm at the same time point, but were only 84.8% identical, corresponding to 50 out of 328 differing nucleotide positions.

Strains from the different Danish outbreaks, such as those centered on Saeby and Holstebro formed clearly delineated clusters in the phylogenetic tree (Fig. 1). *Within* these clusters, there was very low diversity, with pairwise sequence identities ranging from 97% to 100%. However, the diversity *between* the Danish clusters was considerable, and almost as high as that seen when comparing to unrelated viruses from the other investigated countries, with typical between-cluster pairwise sequence identities in the range of 81% to 92%.

Viruses originating from mink sampled in USA (green) and Canada (red) clustered tightly, with pairwise sequence identities of up to 94%. Two sequences originating from mainland Canada grouped together with sequences obtained from a more recent outbreak in Newfoundland [17], differing at only 3–7 nucleotide positions (corresponding to sequence identities of 98% - 99%). Interestingly, both the Danish Saeby cluster and the Finnish sequences (turquois) were closely related to the North American sequences (Fig. 1), suggesting a possible recent common ancestor.

Sequences from Lithuania (pink) and Sweden (orange) grouped together (Fig. 1) and had sequence identities up to 96%. The majority of Greek sequences (aubergine color) clustered with sequences from the Netherlands (blue), but generally, sequences originating from the Netherlands, Italy (purple), Sweden and Poland (brown) were dispersed over several clusters containing a mixture of sequences from different European countries. Spanish sequences (dark grey) from two different farms clustered closely with 98–100% sequence identity, and the closest foreign match to these were to sequences from Finland, but only with a sequence identity of 93%. The wild mink sequences from Iceland (dark green) were most closely related to Swedish sequences from farmed mink, but with only from 93.3% sequence identity. The sequences that resembled the sequences of Latvia (grey) the most were Danish sequences from the Saeby cluster (pairwise sequence identity up to 96%). Finally, two Swedish sequences were found to be very similar (pairwise identity 98%) to sequences originating from the 2015 Danish outbreak cluster on the island Zealand which is situated geographically close to Sweden.

All sequences from this study are available in GenBank with the accession numbers: MG366604-MG366817.

## Discussion

To our knowledge this is the first study of the global diversity of contemporary strains of AMDV. The study showed that partial NS1 sequencing can be used for determining major clusters in AMDV outbreaks. The phylogenetic analysis demonstrated substantial genetic diversity among the sequences collected within each country but also within farms, and confirmed results from previous studies of AMDV diversity based on the partial NS1 gene [6, 10–12, 18, 19]. A possible explanation for the high genetic diversity found in the present study could be that many of the countries from which the samples originate do not have a defined eradication strategy and have enzootic circulation of AMDV, which increases the risk of introduction of different strains. The relatively high level of nucleotide diversity observed here is in agreement with the generally high mutation rate seen in Parvoviruses [2, 12].

Most viral sequences grouped according to the farm they originated from. However, considerable diversity was also observed *within* some farms, suggesting multiple introductions of AMDV. This observation is in agreement with a study by Canuti et al. [17] were 42% of the animals examined for AMDV were infected with more than one viral strain. In countries where no eradication or control measures are carried out, viruses may persist on farms for longer timespans and thereby accumulate higher levels of diversity. The high environmental stability of the virus could compound this problem further. It also seems likely that the fast selection of new breeding stocks from the population within a farm drives the viral evolution even further [17].

The Danish clusters displayed relatively low levels of diversity probably because all the samples originated from contemporary outbreaks, meaning that several animals were sampled around the same period of time and always in connection with outbreaks. This pattern of low diversity also suggested that a single source was the cause of each of the different Danish outbreaks (Saeby, Holstebro and Zeeland, respectively). The diversity among strains from the three Danish clusters cannot be explained by genetic drift and therefore it is obvious that distinct viral strains caused each of these outbreaks [11]. Samples from the other countries included in this study did not originate from outbreaks but were collected from farms having persistently AMDV infected mink and thus exhibited more diversity. This study furthermore indicated that it might be possible to identify transport of virus between countries. For instance, sequences from Finnish mink clustered close to the North American sequences, in agreement with the idea that Finnish minks were probably initially imported from USA and Canada [18]. Our results also indicated movement of viral strains between Greece and the Netherlands and between Lithuania and Sweden – most likely due to trade of live animals. Furthermore, sequences from American mink clustered close to the Danish Saeby strain, which might be explained by the purchase of live mink from USA in the eighties. The AMDV status of the farms and the mink imported at that time were often not known, and shortly after importing these animals, several farms were found positive for AMDV. Similarly, Canadian sequences were closely related to both the American and Saeby cluster strains, an observation which also can be explained by trade of mink from either USA or Denmark to Canada (Mariann Chriél, personal communication). Unfortunately, there were no farm records available to confirm this.

Of particular interest with respect to the recent Danish outbreaks is that that two sequences from a Swedish farm were closely related to viruses from the outbreak on the Danish island Zealand. The pairwise sequence identity equaled that within the Danish Sæby and Holstebro outbreak clusters, thereby indicating the Zealand outbreak could have an epidemiologic link to Sweden. Zealand had been free of AMDV from 1998 to 2015. Since AMDV is a highly resistant virus it is likely that virus can be transferred between mink farms by trade of live, subclinically infected mink, aerosols between adjacent farms, or through passive transmission with persons, wild life or shared farm equipment [5, 20–22].

The sequences originating from the Holstebro outbreak were not closely related to any of the other sequences included in this study and due to their high pairwise sequence identity it was not possible to infer the relationship between them. Thus, the phylogenetic analyses could not provide any additional information on the source of this recent outbreak. The epidemiological data suggested a feed-borne source of the outbreak [11] and the hypothesis was that one of the feed ingredients was contaminated with AMDV originating from the country where the product was processed. Since a limited number samples were analyzed from each country, it cannot be ruled out that the strains responsible for the outbreak originated from countries included in the study in that most countries have a number of different AMDV strains circulating.

Another possible route through which new strains of AMDV could be introduced in Denmark is via AMDV infected wild mink entering the feed producer’s production facilities or feed silos on farms. Only one wild mink from Denmark and sixteen from Iceland were sampled in this study. The viral sequence obtained from the Danish wild mink was very different from the other known AMDV strains in Denmark, suggesting that the wild population is not the source of the AMDV strains circulating in farmed animals. However, more extensive sampling of the Danish wild mink population would be necessary to more conclusively determine whether the wild mink population acts as a reservoir of AMDV. It should be noted that in addition to mink other members of the mustelid species can also be infected with AMDV [23] and that they may thereby pose a threat for the farmed mink, and it could therefore be relevant to sample other species as well [18, 19, 23]. Finally, migration of wild mink between Denmark and Germany could also have led to the introduction of new AMDV strains.

Iceland is the only country in the world that has managed completely to eradicate AMDV from mink farms, although one case of AMDV after the eradication suggested that the virus may still be present in the wild population of mink [8]. In the present study, the sequences obtained from the Icelandic wild mink were most closely related to viruses from Sweden, but the sequence identity was not very high. However, the fact that AMDV was present in the wild mink of Iceland, emphasized the importance of having good biosecurity procedures in place to ensure wild mink from gaining access to farm areas, especially in Iceland and other countries where AMDV is circulating in the wild fauna. Conversely, farmed mink may also act as a reservoir for introduction of AMDV into the wild mink population.

It should be emphasized that the section of the NS1 gene analyzed here represents only 7% of the full genome which has the consequence that the present data set provides limited power to detect differences between individual farms during an outbreak, and to track transmission of virus belonging to the same cluster between farms. In addition to the issue of limited resolution of the phylogeny, a drawback with different partial sequences is that they sometimes result in different and conflicting tree topologies [17, 19]. It has however been shown that by sequencing the entire AMDV genome the phylogenetic resolution is increased to a level where transmission routes between farms can be elucidated [24, 25], however the costs associated with the next generation sequencing (NGS) approach are substantial.

## Conclusion

To the authors knowledge this is the first major study on the global distribution and diversity of AMDV, and these sequence data contribute to a more detailed understanding of the epidemiology of AMDV strains found worldwide. The study revealed that partial NS1 sequencing can be used to distinguish virus strains belonging to major clusters. The clear link between one of the Danish outbreaks and a strain from another country (i.e. Sweden) showed that partial NS1 sequencing can be a useful tool in outbreak tracing, especially in combination with epidemiological data to guide samplings and facilitate interpretation of the phylogenetic analyses. However, it is clear that the additional information from full genome sequencing would allow higher resolution of the phylogenetic analyses.

## Additional file

Additional file 1: Global pholygentic tree of AMDVs. (PDF 1920 kb)

## Abbreviations

AMDV: Aleutian mink disease virus
ELISA: Enzyme-linked immunosorbent assay
NGS: Next generation sequencing
NS1: Nonstructural protein 1
PCR: Polymerase chain reaction
