# Supplementary material for: Global phylogenetic analysis of contemporary aleutian mink disease viruses (AMDVs)
Source: Virol J. 2017 Nov 22;14:231. doi: 10.1186/s12985-017-0898-y (PMC5700682; doi:10.1186/s12985-017-0898-y)
Supplement: Additional file 1: — Global pholygentic tree of AMDVs. (PDF 1920 kb) [file 12985_2017_898_MOESM1_ESM.pdf]

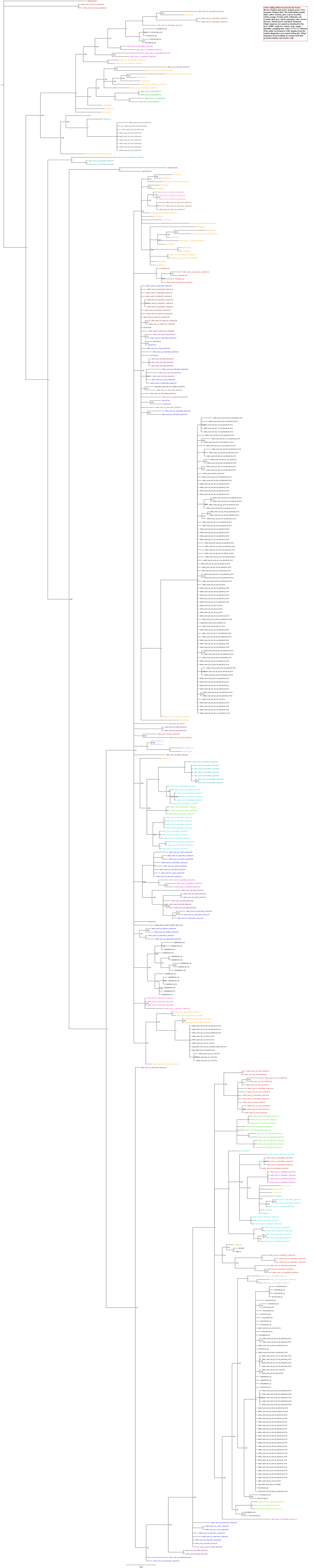
Color coding of the tree given by the Taxon: Brown: Poland, dark green: Iceland, green: USA, turquois: Finland, blue: The Netherlands, purple: Italy, yellow: Estonia, grey: Latvia, lavender: China, orange: Sweden, pink: Lithuania, red: Canada, dark grey: Spain, aubergine color: Greece, Norway: Marine green. and black: Denmark. Single sequences are named as mentioned in the text: AMDV_mink-f/w_country code_unique identifier_sampling date, where “f “or “w” indicates if the mink was farmed or wild. Samples from the routine diagnostics were named with prefix “Diag”. Sequences from GenBank are named with their accession number and country code.
